# Characterization and structure-activity relationships of indenoisoquinoline-derived topoisomerase I inhibitors in unsilencing the dormant *Ube3a* gene associated with Angelman syndrome

**DOI:** 10.1186/s13229-018-0228-2

**Published:** 2018-08-17

**Authors:** Hyeong-Min Lee, Ellen P. Clark, M. Bram Kuijer, Mark Cushman, Yves Pommier, Benjamin D. Philpot

**Affiliations:** 1 Department of Cell Biology and Physiology, University of North Carolina School of Medicine, Neuroscience Research Building, Room 5119 115 Mason Farm Rd., Campus Box 7545, Chapel Hill, NC 27599-7545 USA; 2 Department of Medicinal Chemistry and Molecular Pharmacology, Purdue University School of Pharmacy and the Purdue Center for Cancer Research, West Lafayette, IN USA; 3 Developmental Therapeutics Branch and Laboratory of Molecular Pharmacology, Center for Cancer Research, National Cancer Institute, Bethesda, MD USA; 4 UNC Neuroscience Center, Carolina Institute for Developmental Disabilities, University of North Carolina School of Medicine, Chapel Hill, NC USA

**Keywords:** Angelman syndrome, UBE3A, Topoisomerase I, Topotecan, Indenoisoquinoline, Topoisomerase inhibitor, Indotecan

## Abstract

**Background:**

Angelman syndrome (AS) is a severe neurodevelopmental disorder lacking effective therapies. AS is caused by mutations in ubiquitin protein ligase E3A (*UBE3A*), which is genomically imprinted such that only the maternally inherited copy is expressed in neurons. We previously demonstrated that topoisomerase I (Top1) inhibitors could successfully reactivate the dormant paternal allele of *Ube3a* in neurons of a mouse model of AS. We also previously showed that one such Top1 inhibitor, topotecan, could unsilence paternal *UBE3A* in induced pluripotent stem cell-derived neurons from individuals with AS. Although topotecan has been well-studied and is FDA-approved for cancer therapy, its limited CNS bioavailability will likely restrict the therapeutic use of topotecan in AS. The goal of this study was to identify additional Top1 inhibitors with similar efficacy as topotecan, with the expectation that these could be tested in the future for safety and CNS bioavailability to assess their potential as AS therapeutics.

**Methods:**

We tested 13 indenoisoquinoline-derived Top1 inhibitors to identify compounds that unsilence the paternal allele of *Ube3a* in mouse neurons. Primary cortical neurons were isolated from embryonic day 14.5 (E14.5) mice with a *Ube3a-YFP* fluorescent tag on the paternal allele (*Ube3a*^*m+/pYFP*^ mice) or mice that lack the maternal *Ube3a* allele and hence model AS (*Ube3a*^*m−/p+*^ mice). Neurons were cultured for 7 days, treated with drug for 72 h, and examined for paternal UBE3A protein expression by Western blot or fluorescence immunostaining. Dose responses of the compounds were determined across a log range of drug treatments, and cytotoxicity was tested using a luciferase-based assay.

**Results:**

All 13 indenoisoquinoline-derived Top1 inhibitors unsilenced paternal *Ube3a*. Several compounds exhibited favorable paternal *Ube3a* unsilencing properties, similar to topotecan, and of these, indotecan (LMP400) was the most effective based on estimated E_max_ (maximum response of unsilencing paternal *Ube3a*) and EC_50_ (half maximal effective concentration).

**Conclusions:**

We provide pharmacological profiles of indenoisoquinoline-derived Top1 inhibitors as paternal *Ube3a* unsilencers. All 13 tested compounds were effective at unsilencing paternal *Ube3a*, although with variable efficacy and potency. Indotecan (LMP400) demonstrated a better pharmacological profile of *Ube3a* unsilencing compared to our previous lead compound, topotecan. Taken together, indotecan and its structural analogues are potential AS therapeutics whose translational potential in AS treatment should be further assessed.

**Electronic supplementary material:**

The online version of this article (10.1186/s13229-018-0228-2) contains supplementary material, which is available to authorized users.

## Background

Angelman syndrome (AS) is a severe neurodevelopmental disorder characterized by developmental delay, intellectual disability, speech impairment, seizures, and ataxia [1–5]. AS has a prevalence of 1:15,000 [6, 7], and these individuals need care across their full lifespan, yet no cure currently exists. Thus, it is of great importance to develop treatments for AS. AS is caused by mutation of the ubiquitin protein ligase E3A (*UBE3A*) gene, which is genomically imprinted. Only the maternally inherited copy is expressed in neurons [8], whereas *UBE3A* is biallelically expressed in most other tissues. This neuron-specific imprinting provides insight into why deletions or mutations in the maternal copy of *UBE3A* primarily impact brain function and cause AS. However, the paternal *UBE3A* allele is intact, as demonstrated by biallelic expression in other tissues, raising the possibility that AS could be treated by unsilencing the dormant paternal *UBE3A* allele in neurons.

This led us to try pharmacological approaches to identify small molecules capable of unsilencing the dormant copy of *UBE3A*. In a previous study, we developed a high-content assay to identify small molecules that could unsilence paternal *Ube3a* in mouse primary neurons. In that screen, we used knock-in mice carrying a yellow fluorescent protein (YFP)-tagged *Ube3a* reporter, allowing us to visualize maternal- or paternal-specific expression of *Ube3a*-*YFP* in cultured neurons. As expected, *Ube3a*-*YFP* was expressed in cultured neurons when inherited maternally but was not expressed (silenced) when inherited paternally. We found that topoisomerase I (Top1) inhibitors (e.g., topotecan) could effectively unsilence paternal *Ube3a* in mice [9], raising the possibility that topotecan or similar compounds [10] could become treatments for AS. The translational potential was supported by evidence that topotecan treatment biochemically rescued the function of UBE3A, unsilenced *Ube3a* in vivo in mice, and unsilenced paternal *UBE3A* in induced pluripotent stem cell-derived neurons of AS patients [11].

Topotecan is FDA-approved for the treatment of cancer and is well tolerated in adult and pediatric cancer patients [12–15]. It is also used to treat brain tumors [16, 17]. Topotecan crosses the BBB more readily than many topoisomerase inhibitors [18]. However, active pumps extrude topotecan from the brain, which limits its functional CNS bioavailability [19, 20]. Moreover, topotecan can produce some toxicities [21, 22]. These limitations prompted us to search for novel Top1 inhibitors with better CNS bioavailability and improved safety profiles.

Indenoisoquinoline-derived Top1 inhibitors offer a promising class of compounds for paternal *Ube3a* unsilencing as many of these compounds produce particularly stable Top1 cleavage complexes [23–25], which we have shown are critical for producing paternal *Ube3a* unsilencing [26]. Over 300 indenoisoquinoline derivatives have been tested, some of which are very potent Top1 poisons and show antitumor activity in mouse models [10, 27–31]. These Top1 inhibitors work by blocking the enzymatic activity of Top1 by stabilizing cleavage complexes, which are compound-bound intermediates of Top1-DNA [10, 23–25, 32]. More importantly, when compared to topotecan, indenoisoquinoline-derived Top1 inhibitors demonstrate improved characteristics such as greater chemical stability of these cleavage complexes. In addition, they target a unique DNA sequence for cleavage (indenoisoquinolines --G^↓^C-- vs. topotecan --T^↓^G--) [23–25, 32].

The goal of this study was to establish indenoisoquinoline derivatives that could effectively unsilence paternal *Ube3a*, with the expectation that some of these compounds might prove to be safe and have favorable CNS bioavailability. All of the tested compounds showed a capacity to unsilence the paternal *Ube3a* allele, with several of the compounds exhibiting unsilencing efficacy similar to topotecan. Excitingly, two of the tested indenoisoquinoline derivatives, indotecan (LMP400) and indimitecan (LMP776), are already in clinical trials [33, 34]. The results of our study suggest additional Top1 inhibitors that should be advanced for AS preclinical testing of safety and CNS efficacy.

## Methods

### Animals

All animal experiments were handled with an Institutional Animal Care and Use Committee (IACUC) protocol approved by the University of North Carolina School of Medicine. AS model mice [35] (*Ube3a*^*m−/p+*^) were generated by crossing *Ube3a*^*m+/p−*^ females with wildtype males. Paternal YFP-tagged mice [36] (*Ube3a*^m*+/pYFP*^) were generated by crossing heterozygote *Ube3a-*YFP males with wildtype females. Mice were housed at 12 h:12 h LD and given ad libitum access to water and food. Both male and female mice (embryos) were used in all studies.

### Chemistry

Topotecan was purchased from Cayman Chemicals. Indotecan (LMP400) and indimitecan (LMP776) were obtained from the Developmental Therapeutics Program (DTP) branch, National Cancer Institute. Eleven structural analogues of indotecan and indimitecan, all indenoisoquinoline derivatives, were provided by Dr. Mark Cushman at Purdue University. Syntheses of 12 indenoisoquinolines have been previously described: indotecan (LMP400) and indimitecan (LMP776) [33], DB-III-17, DB-IV-26, DB-IV-50, DB-IV-56, DB-IV-58, DB-V-37, DB-V-41, DB-V-46, and DB-V-47 [37], and MJ-II-66A [30]. MNR-IV-64 was synthesized by the procedure reported for the corresponding *N-4′*-hydroxybutyl analogue [30]. These compounds were selected because they have a wide range of cytotoxicities in cancer cell culture while maintaining some degree of Top1 inhibitory activity. All compounds were stored at − 20 °C before being reconstituted in dimethyl sulfoxide (DMSO) prior to use.

### Cell culture and drug treatment

**Fig. 1:**
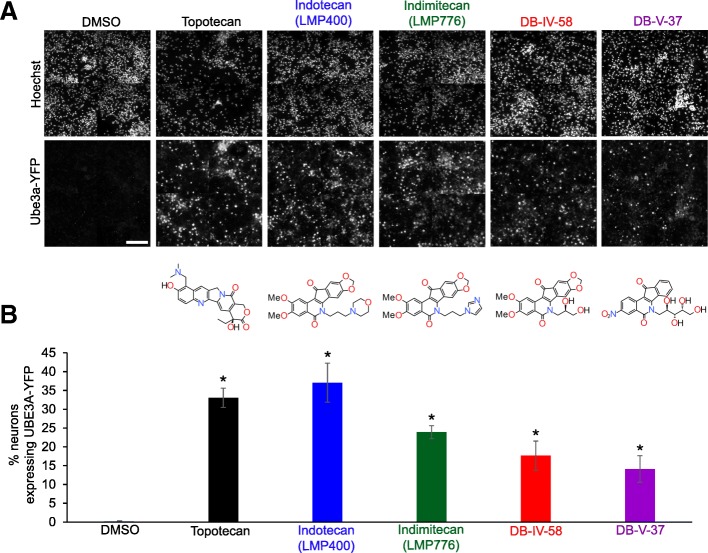
Like the camptothecin-derived topotecan, indenoisoquinoline derivatives can unsilence paternal *Ube3a-YFP* in vitro. **a** Immunofluorescence images of nuclei (Hoeschst stain) and paternal UBE3A-YFP in drug-treated cultured mouse cortical neurons and chemical structures of the compounds. Paternal *Ube3a-YFP* was unsilenced by the indicated drugs [topotecan (0.3 μM), indotecan (0.3 μM), indimitecan (0.3 μM), DB-IV-58 (0.3 μM), or DB-V-37 (0.3 μM)] but not by DMSO vehicle control (scale bar = 100 μm). DB-IV-58 and DB-V-37 are structural analogues of indotecan and indimitecan. **b** Quantitative analysis of neurons expressing unsilenced paternal UBE3A-YFP (*n* = 4 wells in 384-well plate/group, *p < 0.05)

**Fig. 2:**
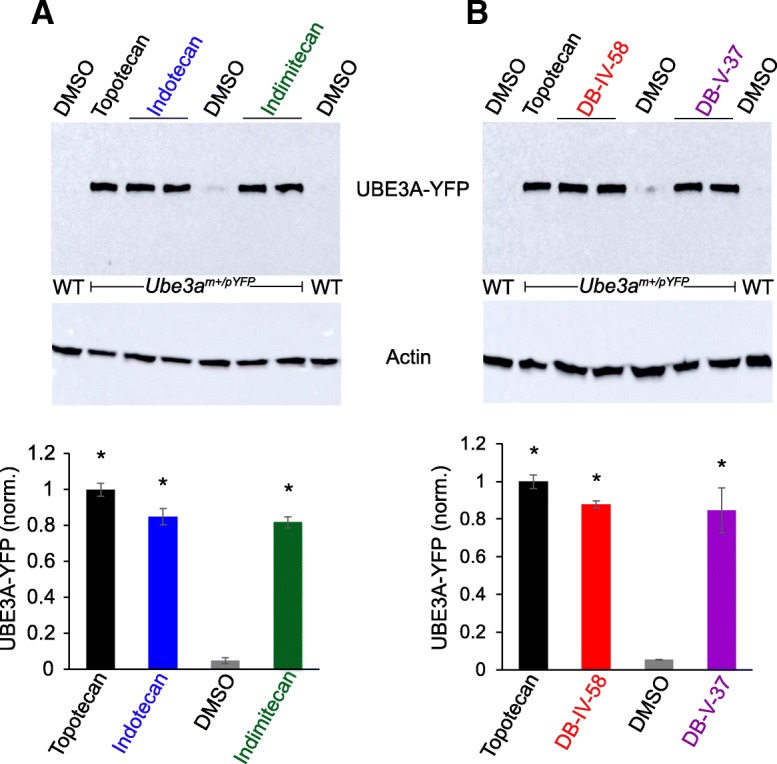
Western blot analysis demonstrating the capacity of indenoisoquinoline derivatives to increase paternal UBE3A-YFP at the protein level in cultured neurons from *Ube3a*^m*+/pYFP*^ mice. **a** Immunoblot and quantification of UBE3A-YFP levels normalized to actin in cultured neurons from wildtype (WT) or *Ube3a*^m*+/pYFP*^ mice treated with DMSO (0.1% vehicle control), topotecan (0.3 μM), indotecan (0.3 μM), or indimitecan (0.3 μM) (*n* = 3/group, *p < 0.05). **b** Immunoblots and quantification of UBE3A-YFP levels normalized to actin in cultured neurons from wildtype (WT) or *Ube3a*^m*+/pYFP*^ mice treated with DMSO (0.1% vehicle control), topotecan (0.3 μM), DB-IV-58 (0.3 μM), or DB-V-37 (0.3 μM) (*n* = 3/group, *p < 0.05)

**Fig. 3:**
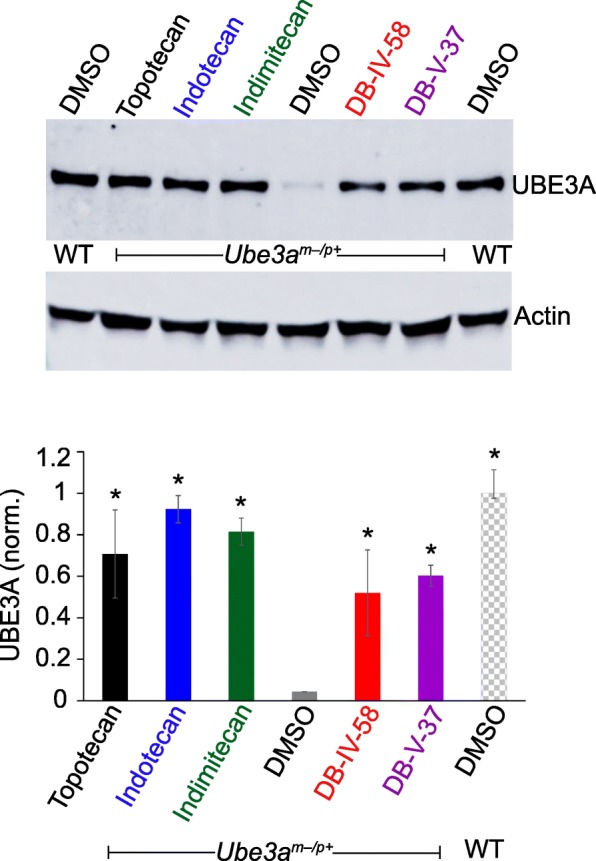
Indenoisoquinoline derivatives unsilence paternal *Ube3a* in AS model mice (*Ube3a*^*m−/p+*^). Immunoblot and quantification of UBE3A levels normalized to actin in cultured neurons from wildtype (WT) or *Ube3a*^*m−p+*^ mice treated with DMSO (0.1% vehicle control), topotecan (0.3 μM), indotecan (0.3 μM), indimitecan (0.3 μM), DB-IV-58 (0.3 μM), or DB-V-37 (0.3 μM) (*n* = 3/group, *p < 0.05)

**Fig. 4:**
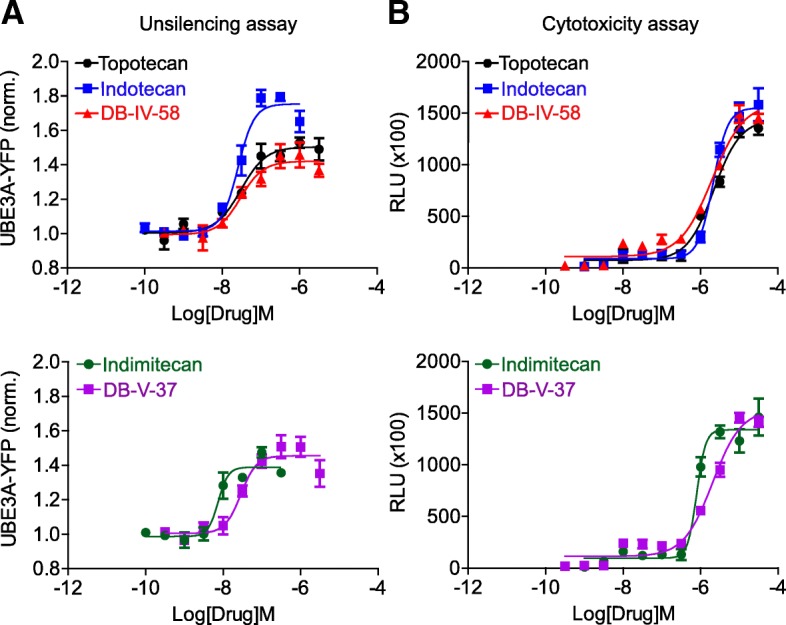
Pharmacological properties of four indenoisoquinoline derivatives in unsilencing paternal *Ube3a-YFP* in vitro. **a** Dose dependence of four indenoisoquinoline derivatives and topotecan in unsilencing of paternal UBE3A-YFP (*n* = 4/group). Estimated potencies and efficacies of the drugs are summarized in Table 1. **b** Dose-dependent cytotoxicities of four indenoisoquinoline derivatives and topotecan (*n* = 4/group). Estimated LC_50_ values are summarized in Table 1

Primary cortical neurons were isolated and cultured using previously described protocols [9]. Briefly, we isolated cortical neurons from embryos (E14.5) carrying paternal *Ube3a-YFP* (*Ube3a*^m*+/pYFP*^) or maternal deletion of *Ube3a* (*Ube3a*^m*−/p+*^). Isolated cortical neurons were plated onto 384-well plates (~ 25,000 cells/well) for high-content imaging and onto 6-well plates (~ 1,000,000 cells/well) for Western blot analysis. Cultured neurons were initially incubated for 7 days, replacing culture medium every 3–4 days. Drugs were freshly prepared in DMSO as a 10-mM stock, unless further dilution in DMSO was necessary. On day 7 (DIV 7), the indicated compounds (topotecan, indotecan, indimitecan, DB-IV-58, and DB-V-37 in Figs. 1, 2, and 3) were directly added at 0.3 μM (final concentration in culture medium) to the neurons for 72 h to allow time for unsilencing of paternal *Ube3a* or *Ube3a*-YFP. For dose dependence and cytotoxicity tests (Fig. 4 and Additional file 1), we used half-log molar drug concentrations, 1 × 10^−10^, 3 × 10^−10^, 1 × 10^−9^, 3 × 10^−9^, 1 × 10^−8^, 3 × 10^−8^, 1 × 10^−7^, 3 × 10^−7^, 1 × 10^−6^, 3 × 10^−6^, 1 × 10^−5^, and 3 × 10^−5^ M.

### Fluorescence immunostaining and high-content imaging

We followed the protocols for fluorescent immunostaining and high-content imaging of cortical neurons as previously described [9, 38]. Briefly, 72 h after drug treatment, the cells were fixed with 4% paraformaldehyde at room temperature for 15 min. After rinsing with phosphate-buffered saline (PBS) three times, the cells were permeabilized with 1% Triton-X 100 in PBS, followed by blocking with 5% NGS and 0.1% Triton-X 100 in PBS at room temperature for 30 min. After blocking, the cells were incubated with primary antibody, rabbit anti-GFP (1:1000, Novus Biologicals), at 4 °C overnight. The cells were then briefly rinsed with PBS followed by incubation with secondary antibody, goat anti-rabbit Alexa Fluor 488 (1:200, Thermo Fisher/Invitrogen), at room temperature. One hour after secondary antibody incubation, the cells were rinsed with PBS and fluorescent images were acquired using a BD Pathway 855 bioimager. The acquired images were processed by CellProfiler [39] to count the number of positive cells and measure fluorescent intensity (Additional file 2). To determine the percentage of neurons expressing paternal *Ube3a-YFP*, we counted the total number of cells (Hoechst) and YFP-positive cells, and the number of YFP-positive cells was divided by the total number of cells. Fluorescence intensity was measured in neurons expressing unsilenced paternal *Ube3a-YFP* and normalized to vehicle control.

### Cytotoxicity test

Toxicity of the compounds was tested in cultured cortical neurons in vitro. Using Cyto Tox-Glo assay (Promega), we followed the manufacturer’s protocol to measure luminescence proportional to the number of dead vs. live cells. Briefly, 72 h after drug treatment, we directly added AAF-Glo substrates into the drug-treated (or 0.1% DMSO vehicle-treated) neurons and incubated them at room temperature for 15 min. We then measured luminescence produced by dead-cell protease activity.

### Western blot analysis

We followed the same procedures of Western blot analysis as previously described [9, 38]. In brief, 72 h after drug treatment, we collected the cultured neurons from 6-well plates and extracted total protein with protein extraction buffer. Bradford assay was performed to measure protein concentration, and 30 μg of total protein was loaded for Bis-polyacrylamide gel electrophoresis (Bio-Rad). Electrophoresed proteins were transferred to nitrocellulose membrane (0.45 μm, Bio-Rad). The membranes were blocked with 5% non-fat milk in TBS-T at room temperature for 30 min followed by overnight 4 °C incubation with primary antibodies (rabbit anti-GFP, 1:1000, Novus Biologicals; rabbit anti-Ube3a, 1:1000, Bethyl Lab; mouse anti-actin; 1:5000, Sigma). The next day, the membranes were rinsed with TBS-T three times and incubated with HPR-conjugated secondary antibodies for 1 h at room temperature (goat anti-rabbit, 1:1000, Vector Lab or goat anti-mouse, 1:1000, Vector Lab). Following secondary antibody incubation, the membranes were rinsed with TBS-T at room temperature for 1 h (4–5 times) and ECL substrates (Bio-Rad) were used to visualize immunostaining using an Amersham Imager 600 (AI600, GE Life Sciences).

### Statistical analysis

One-way ANOVA was performed to determine significant differences in unsilencing paternal *Ube3a*-YFP or *Ube3a*, followed by Dunnett’s multiple comparison test. Two-way ANOVA was performed to determine changes in EC_50_, E_max_, and LC_50_ (Table 1), with comparisons to topotecan made using a Bonferroni correction for multiple comparisons.

**Table 1:** Summary of efficacies and potencies of topotecan and indenoisoquinoline derivatives

Compound	EC_50_ [M]	E_max_	LC_50_ [M]
Topotecan	3.04E−08 (± 3.40E−09)	1.51 (± 0.11)	2.30E−06 (± 2.01E−08)
Indotecan	2.56E−08 (± 1.05E−09)*	1.78 (± 0.13)*	2.01E−06 (± 1.59E−07)
Indimitecan	7.47E−09 (± 7.49E−10)*	1.47 (± 0.10)	7.89E−07 (± 3.43E−08)*
DB-IV-58	3.10E−08 (± 9.75E−09)	1.45 (± 0.12)	2.01E−06 (± 1.09E−07)
DB-V-37	2.99E−08 (± 8.29E−09)	1.48 (± 0.08)	2.02E−06 (± 1.02E−07)

EC_50_, E_max_, and LC_50_ are estimated from data presented in Fig. 4. Significance was tested by two-way ANOVA, followed by a Bonferroni correction for multiple comparisons to determine significant differences (*p < 0.05) from topotecan-treated neurons

## Results

### Indenoisoquinoline-derived topoisomerase I inhibitors effectively unsilence paternal Ube3a

The goal of this study was to identify novel Top1 inhibitors as potential AS therapeutics. We chose to focus on the compounds indotecan (LMP400), indimitecan (LMP776), and their analogues because indotecan and indimitecan recently completed phase I clinical trial testing at the National Institutes of Health (ClinicalTrials.gov ID: NCT01051635). Moreover, indotecan exhibits favorable CNS penetration [40]. Thus, these drugs have already undergone a certain degree of preclinical safety testing, which could expedite clinical development if warranted.

As we anticipated, paternal *Ube3a-YFP* was not expressed at appreciable levels in cultured neurons in the presence of 0.1% DMSO (vehicle control). On the other hand, topotecan (positive control) unsilenced paternal *Ube3a-YFP* as previously reported [9] (Fig. 1a). Four indenoisoquinoline-derived compounds (indotecan, indimitecan, DB-IV-58, and DB-V-37) also successfully demonstrated unsilencing of paternal *Ube3a-YFP* in our reporter mouse (Fig. 1a). We quantified the number of Hoechst-positive cells expressing paternal *Ube3a-YFP* above a defined threshold and found that few (0.21 ± 0.17%) DMSO-treated neurons expressed *Ube3a-YFP* above threshold (Fig. 1b). In contrast, a significant (*p* < 0.05) number of neurons expressed paternal *Ube3a-YFP* when cultures were treated with topotecan (33.0 ± 2.56%), indotecan (37.1 ± 5.19%), indimitecan (23.9 ± 1.72%), DB-IV-58 (17.7 ± 3.87%), or DB-V-37 (14.1 ± 3.55%) (Fig. 1b). The number of cells expressing paternal *Ube3a-YFP* was similar between indotecan- and topotecan-treated neurons at a dose of 0.3 μM. At this same concentration (0.3 μM), fewer neurons treated with indimitecan, DB-IV-58, or DB-V-37 expressed paternal *Ube3a-YFP* compared to topotecan-treated neurons (*p* < 0.05 compared to topotecan-treated neurons).

To validate the paternal *Ube3a-YFP* unsilencing and definitively rule out the possibility of fluorescence artifacts (e.g., intrinsic fluorescence in compounds), we performed Western blot analysis using primary cultured cortical neurons. No UBE3A-YFP protein was expressed in wildtype neurons (negative control), whereas paternal UBE3A-YFP was marginally detectable in DMSO-treated cells from *Ube3a*^*m+/pYFP*^ mice, possibly due to contamination from glial cells that express *Ube3a* biallelically or to very modest expression of paternal *Ube3a* expressed during early stages of development. Topotecan, indotecan, indimitecan, DB-IV-58, and DB-V-37 treatments led to paternal UBE3A-YFP protein production (Fig. 2). Normalized fold changes in unsilenced UBE3A-YFP were comparable in all five tested drugs (bottom panels in Fig. 2a, b).

Although unlikely, we wanted to rule out the possibility that the unsilencing was an artifact of the *Ube3a-YFP* knock-in. Thus, we tested the drug effects in AS model mice that lack the maternal *Ube3a* allele (*Ube3a*^*m−/p+*^ mice) (Fig. 3). There was little paternal UBE3A protein in DMSO-treated *Ube3a*^*m−/p+*^ neurons compared to *Ube3a*^*m+/p+*^ (wildtype) neurons. Topotecan, indotecan, indimitecan, DB-IV-58, and DB-V-37 treatments result in a high level of paternal UBE3A protein compared to DMSO-treated neurons. These data confirm the ability of the tested indenoisoquinolines to unsilence paternal *Ube3a*.

### Pharmacological profiling of indenoisoquinoline Top1 inhibitors in cultured cortical neurons in vitro

Once we confirmed the unsilencing effects, we performed pharmacological profiling of indotecan, indimitecan, and their analogues for structure-activity relationships in order to identify more effective unsilencers. We performed dose-response experiments for all tested compounds (Fig. 4a and Additional file 1). These experiments identified indotecan as the most efficacious of all of drugs tested, with the potency (EC_50_) of indotecan being significantly better than topotecan (Table 1; *p < 0.05). This suggests indotecan may have potential as a possible AS therapeutic. Indimitecan is less likely as a candidate therapeutic, because although it exhibited good potency in a nanomolar range and had similar efficacy as topotecan (Fig. 4a and Table 1, *p < 0.05), it likely has a low therapeutic index (discussed below). We also examined 11 structural analogues. Among 11 compounds, we found two of them, DB-IV-58 and DB-V-37, with similar efficacy and potency to topotecan (Fig. 4a and Table 1). However, they were less effective than indotecan.

Indenoisoquinoline derivatives were primarily designed to inhibit cancer cell growth. Because our goal is to repurpose these drugs to unsilence a CNS target, it was necessary to test whether the compounds would be deleterious in neuronal cells. Toxicity testing of four indenoisoquinoline-derived compounds (indotecan, DB-IV-58, DB-V-37, and indimitecan) in the cultured neurons revealed that the cytotoxicity of the first three drugs (indotecan, DB-IV-58, and DB-V-37) was similar to that of topotecan. On the other hand, indimitecan exhibited toxicity at a significantly lower concentration than topotecan (Fig. 4b and Table 1, *p < 0.05), suggesting that it might have a low therapeutic index. Lastly, we also tested dose dependency of nine additional analogues (Additional file 1 and Additional file 3: Table S1). All nine compounds could unsilence paternal *Ube3a-YFP* to a certain degree. However, their effectiveness did not exceed that of indotecan. Three compounds (DB-IV-50, DB-IV-56, and MNR-IV-64) share similar pharmacological profiles, as their efficacy and potency were similar. However, their effectiveness of unsilencing paternal *Ube3a-YFP* was less than our lead compounds. Six compounds (DB-III-17, DB-IV-26, DB-V-41, DB-V-46, DB-V-47, and MJ-II-66A) unsilenced paternal *Ube3a-YFP* to a certain degree; however, they showed a very limited range of doses that produce *Ube3a-YFP* unsilencing before showing toxicities (e.g., an unfavorable therapeutic index). Because of the limited dose ranges, their EC_50_ values were not clearly determined (Additional file 3: Table S1). These data together suggest that, compared to topotecan, indotecan has a higher potency and efficacy, but similar toxicity profile, in its ability to unsilence the dormant paternal *Ube3a* allele in neurons.

## Discussion

The goal of this study was to explore indenoisoquinoline derivatives as possible AS therapeutics by characterizing their effects on *Ube3a* unsilencing in mouse cortical neurons in vitro. Here, we identify indotecan (LMP400) as a potential AS therapeutic agent that warrants further examination in vivo for CNS bioavailability and safety.

The unique expression of *UBE3A* governed by genomic imprinting provides a therapeutic opportunity for AS by reactivating the paternal *UBE3A* allele [8, 9, 11]. Our research team previously reported that topoisomerase I inhibitors can reactivate the dormant *UBE3A* allele, providing the first proof of concept of pharmacological reactivation of paternal *UBE3A* as a potential therapeutic intervention for AS [9]. Because of the anticancer activity of Top1 inhibitors, many derivatives that overcome the limitations of camptothecins [10, 41, 42] have been synthesized for clinical development. One of these, topotecan, is FDA-approved for ovarian and lung cancers [43], while another, irinotecan, is approved for colon cancers [44]. However, these compounds may have limited clinical potential for treating AS. For example, topotecan has several flaws such as decreased bioavailability due to plasma protein binding of the lactone hydrolysis product, removal from cells by drug efflux transporters, and long infusion times necessitated by relatively low stability of the ternary drug-DNA-enzyme cleavage complexes [10, 18]. These limitations prompted us to search for novel Top1 inhibitors as potential AS therapeutics, with the expectation that lead candidates could then be vetted for having improved CNS bioavailability and better safety profiles. For these studies, we focused on indenoisoquinoline-derived Top1 inhibitors that might overcome the limitations of topotecan [10]. Of many indenoisoquinoline-derived Top1 inhibitors, indotecan and indimitecan were selected to examine their unsilencing effects on paternal *Ube3a* because of their similar ability to effectively inhibit almost 100% of Top1 enzymatic activity [33] and recent completion of phase I clinical trials (ClinicalTrials.gov ID: NCT01051635).

Here, we demonstrate that indenoisoquinoline-derived Top1 inhibitors are potent *Ube3a* unsilencers, with different unsilencing properties. Of the compounds we tested in vitro, indotecan (LMP400) [10, 34] appears to have more favorable paternal *Ube3a* unsilencing properties than topotecan. While both topotecan and indotecan exhibit CNS penetrance [18, 40], there are not yet data available to directly compare their relative CNS bioavailability; there is a need to carefully establish the CNS bioavailability of indotecan. One potential advantage of indotecan is that it is not a substrate for the transporters ABCG2 and MDR1 [32], suggesting that it may stay longer in the CNS than topotecan because transporters extrude topotecan from the brain [19]. Moreover, although the cytotoxicity profile of indotecan is similar to that of topotecan, its efficacy and potency are better than topotecan. Indimitecan (LMP776) [10] appears to be more toxic than topotecan, while the three other indenoisoquinoline derivatives (indotecan, DB-IV-58, and DB-V-37) showed similar cytotoxicity to topotecan in our cultured cortical neurons. In addition, the efficacy of indimitecan is lower than that of topotecan. The structural differences in indenoisoquinoline-derived compounds are responsible for their different unsilencing effects. The only structural difference between indotecan and indimitecan is in the side chain which is appended to the heterocyclic system that intercalates in the DNA break generated by Top1 [10, 23]. These characteristics will be important considerations for the future design of paternal *UBE3A* unsilencers. Although indotecan exhibits better efficacy and potency than topotecan, the similar cytotoxicity of the two drugs must be considered for in vivo applications. Importantly, the DNA cleavage complex patterns of indotecan are different from topotecan in a manner that may confer some important advantages for clinical use. Indenoisoquinolines such as indotecan produce more stable cleavage complexes than camptothecins such as topotecan [45], which, based on the mechanism of *Ube3a* unsilencing [26], should enhance *Ube3a* unsilencing as we observed. Moreover, after drug removal, the Top1-DNA complexes induced by indenoisoquinolines persist under conditions where camptothecin-induced Top1-DNA complexes completely reverse [45]. This observation further suggests that the similar cytotoxicity of indotecan might be offset by the potential for a briefer treatment regimen in vivo, which remains to be addressed. Known off-target effects for topoisomerase inhibitors generally lead to the transient downregulation of long genes [11, 46, 47]. Genome-wide analyses are necessary to reveal all potential off-target effects for indenoisoquinolines (e.g., indotecan). Alternatively, an in silico analysis using SEA (similarity ensemble approach; http://sea.bkslab.org) enables us to predict off-targets. SEA analysis revealed that indotecan possesses 55 potential off-targets, including aurora kinase A, aurora kinase B, and acetylcholinesterase. Regardless of off-target effects, clinical trials have demonstrated that, at least at the concentrations examined, indotecan is well tolerated in a clinical population [34]. However, it still remains to be addressed whether off-target effects arise at the concentration at which indotecan is effective, as we reported that indotecan has a very low EC_50_ of ~ 26 nM to produce *Ube3a* unsilencing.

We tested 11 structural analogues of indotecan/indimitecan for their ability to unsilence paternal *Ube3a*, and these compounds could be roughly categorized based on their ability to inhibit Top1 in cell-free assays: those compounds (DB-IV-26, DB-IV-50, DB-IV-56, DB-IV-58, DB-V-37, and DB-V-41) that have 100% of the ability of camptothecin to stabilize the ternary drug-DNA-Top1 cleavage complexes [37, 48], those compounds (DB-V-46, DB-V-47, and MNR-IV-64) that inhibit between 50 and 75% of Top1 [37], and those compounds (MJ-II-66A) that inhibit between 20 and 50% of Top1 [30]. We also tested DB-III-17, as this is an intermediate compound for synthesizing or modifying the analogues. The compounds with lower Top1 inhibitory activities (DB-III-17, DB-V-46, DB-V-47, and MJ-II-66A) showed a very limited therapeutic index. Their ambiguous EC_50_ values were mainly due to limited effective dose ranges. Although we did not test their cytotoxicities in our cultured cortical neurons, we expect that they are more toxic than topotecan because we could not measure the fluorescence intensity in unsilenced UBE3A-YFP protein, possibly due to cell death produced at concentrations > 1 μM. On the other hand, we observed similar efficacy and potency of three compounds (DB-IV-50, DB-IV-56, and MNR-IV-64) with between 75 and ~ 100% Top1 inhibitory activities relative to camptothecin, but their effectiveness seems to be less than those of indotecan or topotecan. Interestingly, although DB-IV-26 and DB-V-41 have ~ 100% Top1 inhibitory activity, their EC50 values were also ambiguous due to limited effective dose ranges. Since the cleavage complexes are critical for producing paternal *Ube3a* unsilencing [26], we suspect that their cleavage complexes may not be stable enough to produce paternal *Ube3a* unsilencing. More importantly, the various hydroxylated side chains contribute to differences in the pharmacological action in *Ube3a* unsilencing. For example, the compounds with lower Top1 inhibitory activities (DB-III-17, DB-V-46, DB-V-47, and MJ-II-66A) either lack the hydroxylated side chains that potentially serve as hydrogen-bond acceptors/donors that enable Top1 inhibitory activities and cytotoxicity at physiological pH [49] or the hydroxylated side chain is cyclic. On the other hand, other compounds possessing dimethoxy or methylenedioxy groups, and/or straight hydroxylated side chains, which appear to be the main contributors to Top1 inhibitory activity and cytotoxicity, effectively unsilence paternal *Ube3a*.

Taken together, our study suggests that clinical development of paternal *Ube3a* unsilencers will require optimization of Top1 inhibition and cytotoxicity through modulating chemical characteristics, including the length of the side chains. Although in vivo assays are necessary to further evaluate the unsilencing effects of indenoisoquinolines, this study provides a framework for developing novel AS therapies using different classes of Top1 inhibitors.

## Conclusions

Angelman syndrome is a neurodevelopmental disorder without effective therapeutic interventions. However, pharmacological restoration of the epigenetically silenced copy of *UBE3A* could be one promising approach. Pharmacological inhibition of topoisomerase I (Top1) leads to re-expression of the dormant *UBE3A* allele. Here, we provided pharmacological profiles of indenoisoquinoline-derived Top1 inhibitors as *Ube3a* unsilencers in mouse neurons to identify potential AS clinical candidates. Our data suggest that a number of indenoisoquinolines, and in particular indotecan (LMP400), are potent unsilencers of the paternal *Ube3a* allele and should be further assessed in vivo for their translational potential.

## Additional files

Additional file 1: Chemical structures of nine analogues of indotecan/indimitecan and their pharmacological properties in unsilencing of paternal UBE3A-YFP in vitro. A. DB-III-17, B. DB-IV-26, C. DB-IV-50, D. DB-IV-56, E. DB-V-41, F. DB-V-46, G. DB-V-47, H. MJ-II-66A, I. MNR-IV-64. Estimated potencies and efficacies of the drugs are summarized in Additional file 3: Table S1. (DOCX 13 kb)

Additional file 2: Image analysis in CellProfiler. Morphological changes in the neurons were assessed by nuclear structure of Hoechst-stained neurons. A Immunofluorescence images of Hoechst-stained neurons obtained using a BD Pathway 855 high-content imager (top). The size (between 18 and 40 pixel units) and intensity (threshold range between 0.005 and 1) of stained nuclei were used to segregate putative viable cells recognized as objects (middle, green) from clumped or dead cells (middle, red). Recognized objects were further processed based on size, intensity, and shape (round) (bottom, individual objects were assigned colors by CellProfiler to allow them to be better visualized). No objects were identified in the neurons treated with 10 μM indotecan, as no nuclei met the criteria of immunofluorescence size and intensity. B Quantitative analysis of identified objects. The average numbers of objects were comparable between neurons treated with DMSO (0.1% vehicle control) and indotecan (0.01 μM and 0.3 μM)-treated neurons. (PDF 338 kb)

Additional file 3: **Table S1.** Potency and efficacy of nine analogues (PDF 270 kb)

## Abbreviations

AAF-Glo: Alanyl-alanyl-phenylalanyl-aminoluciferin
AS: Angelman syndrome
BBB: Blood-brain barrier
CNS: Central nervous system
DIV: Day in vitro
FBS: Fetal bovine serum
LMP: Laboratory of Molecular Pharmacology at National Cancer Institutes
NGS: Normal goat serum
PBS: Phosphate buffer saline
PBS-T: Phosphate buffer saline Triton X-100
Ube3a: Ubiquitin protein ligase E3A
*Ube3a*^*m−/p+*^: Maternal deletion of ubiquitin protein ligase E3A and intact paternal Ube3a
*Ube3a*^m *+/pYFP*^: Intact maternal Ube3a and paternal yellow fluorescence protein (YFP)-tagged Ube3a
