# Supplementary material for: Characterization and structure-activity relationships of indenoisoquinoline-derived topoisomerase I inhibitors in unsilencing the dormant Ube3a gene associated with Angelman syndrome
Source: Mol Autism. 2018 Aug 17;9:45. doi: 10.1186/s13229-018-0228-2 (PMC6098585; doi:10.1186/s13229-018-0228-2)
Supplement: Supplementary file 1 — Chemical structures of nine analogues of indotecan/indimitecan and their pharmacological properties in unsilencing of paternal UBE3A-YFP in vitro. A. DB-III-17, B. DB-IV-26, C. DB-IV-50, D. DB-IV-56, E. DB-V-41, F. DB-V-46, G. DB-V-47, H. MJ-II-66A, I. MNR-IV-64. Estimated potencies and efficacies of the drugs are summarized in Additional file 3: Table S1. (DOCX 13 kb) [file 13229_2018_228_MOESM1_ESM.docx]

**Additional file 3: Table S1** Potency and efficacy of 9 analogues

| Compound | EC_50_ [M] | E_max_ |
| --- | --- | --- |
| **DB-III-17** | 3.34E-08 (ambiguous) | 1.26 (±0.08) |
| **DB-IV-26** | 5.56E-07 (ambiguous) | 1.31 (±0.15) |
| **DB-IV-50** | 1.21E-07 (±2.07E-09) | 1.39 (±0.07) |
| **DB-IV-56** | 1.33E-07 (±3.03E-09) | 1.22 (±0.04) |
| **DV-V-41** | 1.19E-08 (ambiguous) | 1.51 (±0.12) |
| **DV-V-46** | 3.67E-08 (ambiguous) | 1.50 (±0.11) |
| **DV-V-47** | 3.39E-07 (ambiguous) | 1.20 (±0.16) |
| **MJ-II-66A** | 2.68E-08 (ambiguous) | 1.20 (±0.05) |
| **MNR-IV-64** | 6.34E-07 (±6.41E-08) | 1.37 (±0.09) |

EC_50_ and E_max_ are estimated in Supplementary Information 1.
