# Supplementary figures and images for: Characterization and structure-activity relationships of indenoisoquinoline-derived topoisomerase I inhibitors in unsilencing the dormant Ube3a gene associated with Angelman syndrome
Source: Mol Autism. 2018 Aug 17;9:45. doi: 10.1186/s13229-018-0228-2 (PMC6098585; doi:10.1186/s13229-018-0228-2)

# Supplementary Information 1

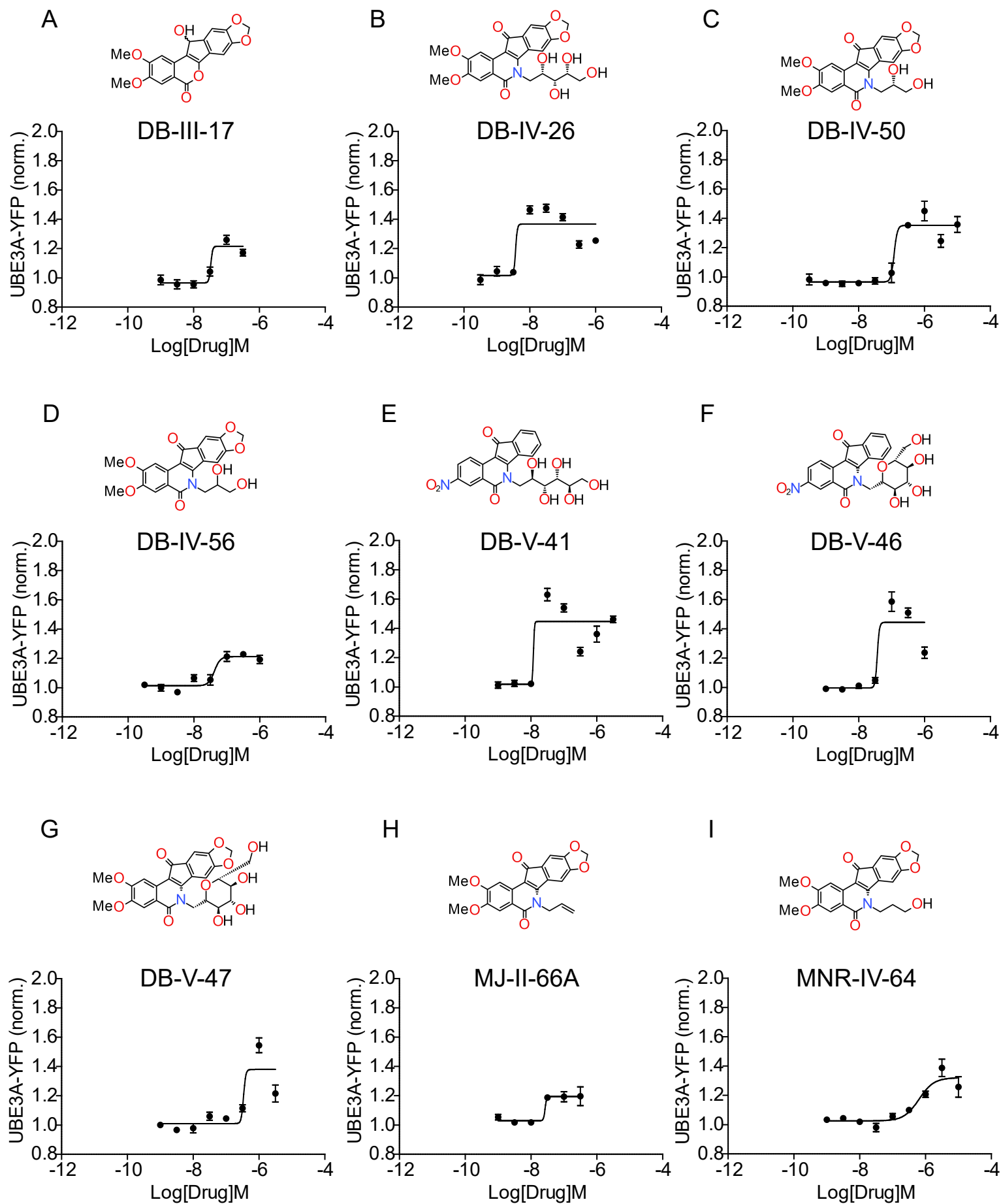

Supplement: Supplementary file 2 — Image analysis in CellProfiler. Morphological changes in the neurons were assessed by nuclear structure of Hoechst-stained neurons. A Immunofluorescence images of Hoechst-stained neurons obtained using a BD Pathway 855 high-content imager (top). The size (between 18 and 40 pixel units) and intensity (threshold range between 0.005 and 1) of stained nuclei were used to segregate putative viable cells recognized as objects (middle, green) from clumped or dead cells (middle, red). Recognized objects were further processed based on size, intensity, and shape (round) (bottom, individual objects were assigned colors by CellProfiler to allow them to be better visualized). No objects were identified in the neurons treated with 10 μM indotecan, as no nuclei met the criteria of immunofluorescence size and intensity. B Quantitative analysis of identified objects. The average numbers of objects were comparable between neurons treated with DMSO (0.1% vehicle control) and indotecan (0.01 μM and 0.3 μM)-treated neurons. (PDF 338 kb) [file 13229_2018_228_MOESM2_ESM.pdf]

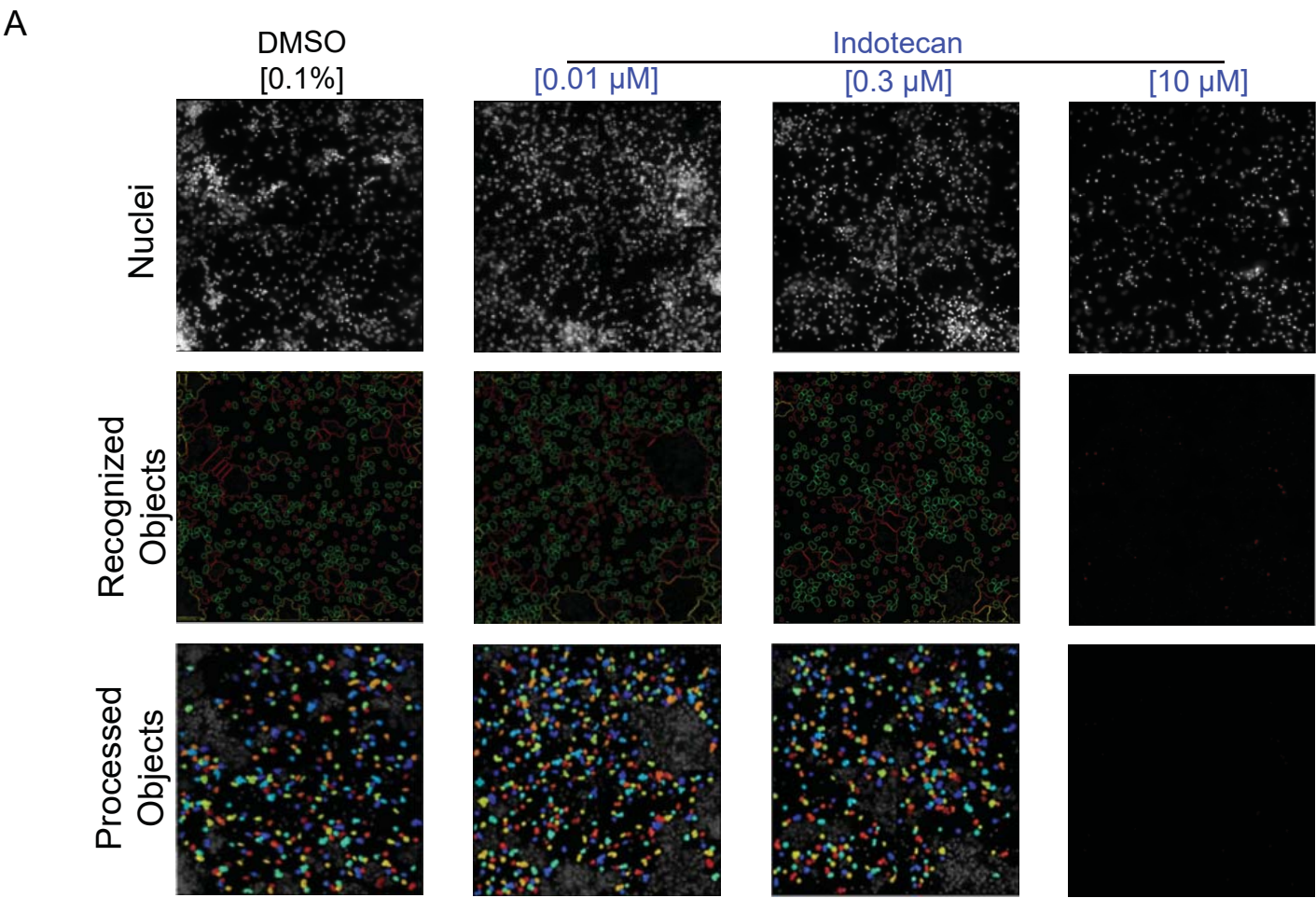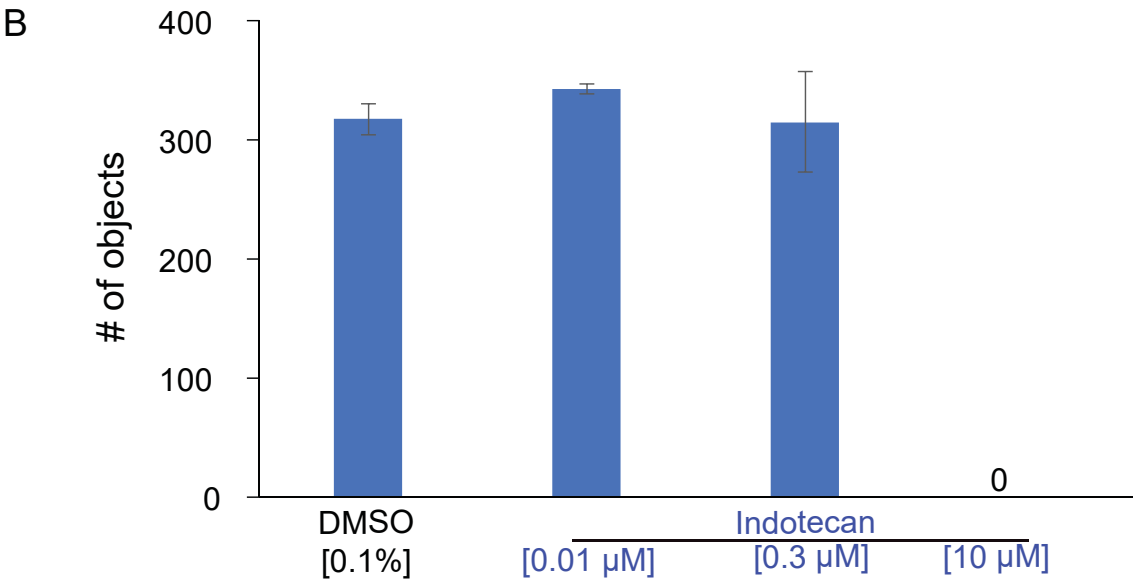

Supplement: Supplementary file 3 — Table S1. Potency and efficacy of nine analogues (PDF 270 kb) [file 13229_2018_228_MOESM3_ESM.pdf]
